# No Routine Postoperative Head CT following Elective Craniotomy – A Paradigm Shift?

**DOI:** 10.1371/journal.pone.0153499

**Published:** 2016-04-14

**Authors:** Ralph T. Schär, Michael Fiechter, Werner J. Z'Graggen, Nicole Söll, Vladimir Krejci, Roland Wiest, Andreas Raabe, Jürgen Beck

**Affiliations:** 1 Department of Neurosurgery, Inselspital, Bern University Hospital, University of Bern, Bern, Switzerland; 2 Department of Anesthesiology, Inselspital, Bern University Hospital, University of Bern, Bern, Switzerland; 3 Department of Neurology, Inselspital, Bern University Hospital, University of Bern, Bern, Switzerland; 4 Department of Neuroradiology, Inselspital, Bern University Hospital, University of Bern, Bern, Switzerland; Universita' degli Studi di Napoli Federico II, ITALY

## Abstract

**Introduction:**

Patient management following elective cranial surgery often includes routine postoperative computed tomography (CT). We analyzed whether a regime of early extubation and close neurological monitoring without routine CT is safe, and compared the rate of postoperative emergency neurosurgical intervention with published data.

**Methods:**

Four hundred ninety-two patients were prospectively analyzed; 360 had supra- and 132 had infratentorial lesions. Extubation within one hour after skin closure was aimed for in all cases. CT was performed within 48 hours only in cases of unexpected neurological findings.

**Results:**

Four-hundred sixty-nine of the 492 patients (95.3%) were extubated within one hour, 20 (4.1%) within 3 hours, and three (0.6%) within 3 to 10 hours. Emergency CT within 48 hours was performed for 43/492 (8.7%) cases. Rate of recraniotomy within 48 hours for patients with postoperative hemorrhage was 0.8% (n = 4), and 0.8% (n = 4) required placement of an external ventricular drain (EVD). Of 469 patients extubated within one hour, 3 required recraniotomy and 2 required EVD placements. Of 23 patients with delayed extubation, 1 recraniotomy and 2 EVDs were required. Failure to extubate within one hour was associated with a significantly higher risk of surgical intervention within 48 hours (rate 13.0%, p = 0.004, odds ratio 13.9, 95% confidence interval [3.11–62.37]).

**Discussion:**

Early extubation combined with close neurological monitoring is safe and omits the need for routine postoperative CT. Patients not extubated within one hour do need early CT, since they had a significantly increased risk of requiring emergency neurosurgical intervention.

**Trial Registration:**

ClinicalTrials.gov NCT01987648

## Introduction

Postoperative patient management following elective cranial surgery varies substantially between different neurosurgical institutions. The common objective in this crucial period is to avoid or detect any early postoperative complications such as intracranial bleeding, ischemia, or brain swelling. Since the introduction of computed tomography (CT) in the 1970s, postoperative head CT within the first hours after neurosurgery has been advocated [1]. These imaging studies are often ordered even in the absence of unexpected neurological findings in order to rule out complications. In many departments patients are not transferred to the wards until they have been “cleared” by CT scanning. This practice of routine head CT scanning has not been substantiated by any prospective evidence, but is perpetuated by common procedural standards and training background of the neurosurgeons [2, 3]. However, there is growing evidence from retrospective series that routine head CT may not be necessary after neurosurgical cranial procedures [2, 4]. Results of recent studies and clinical reasoning argue that repetitive neurological examination and surveillance is key for detection of complications with the need for return to the operating room (OR). Early termination of anesthesia and early extubation is, of course, mandatory for a thorough neurological examination. Today most neurosurgical patients are awakened directly postoperatively in the OR for clinical assessment. Still, some institutions—at least within Europe—prefer a delayed extubation with parameter focused monitoring on the intensive care unit (ICU) over an early extubation in the OR with clinical-neurological monitoring of the awakened patient. The concerns for latter strategy may originate from a fear of too much cardiopulmonary and metabolic distress to the just trephined patient caused by an immediate (“forced”) awakening and extubation with potential sequelae (e.g. postoperative hemorrhage, brain swelling). No evidence from prospective studies exist to support these assumptions.

Therefore we prospectively analyzed a strategy of early extubation without routine head CT after elective cranial neurosurgical procedures. The hypothesis was that the early extubation with dedicated neurological monitoring and no routine head CT strategy provides sufficient patient safety as compared with reported data in the literature.

## Materials and Methods

### Study design

This study was designed as a non-randomized, prospective observational, single center, cohort study. Written informed consent was obtained from all patients. Both the local ethics committee and the institutional review board (IRB) of our University Hospital approved this trial and the means of consent. The study was registered at ClinicalTrials.gov (NCT01987648) and conducted according to GCP guidelines and the Declaration of Helsinki.

### Inclusion criteria

**Fig 1:**
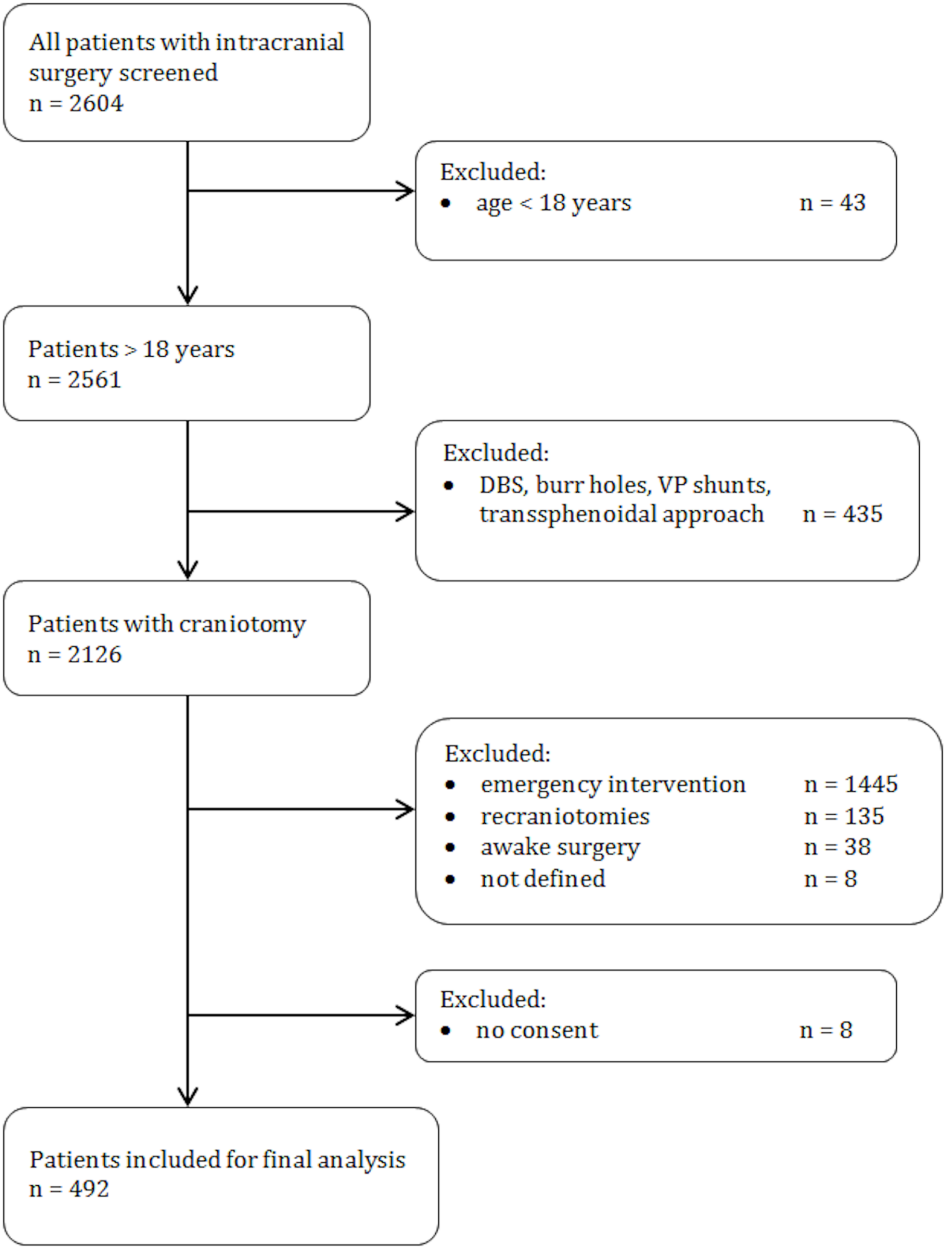
Flow diagram of patients included.

For the period from November 2011 to March 2014 all cranial neurosurgical procedures performed at our University Hospital were prospectively screened. All elective craniotomy cases performed on adults older than 18 years of age were included for final analysis. Patients with a history of prior cranial surgery, procedures for infection, burr hole cases, procedures with transsphenoidal approaches, awake surgery (these patients were not intubated at the time of patient recruitment), or emergency craniotomies were excluded. We intended to assess the first 500 patients included. Of these, 8 patients declined to sign the general consent form and were therefore not included for final analysis (Fig 1).

A total of 2604 patients were screened during the study period, and 492 were included for the final analysis.

### Endpoints

The primary endpoint of this study was return to the OR or ventriculostomy outside the OR due to intracranial hemorrhage, edema, or acute obstructive hydrocephalus within 48 hours. Secondary endpoints were time to extubation after surgery, the rate of ordered emergency head CT scans within 48 hours postoperatively, timing of CT imaging after surgery, duration of surgery (defined as the time in minutes from skin incision to skin closure), and mortality within 30 days after elective craniotomy.

### Data collection

Patient data as defined in a case report form included patient demographics, American Society of Anesthesiologists (ASA) classification of Physical Status Score, preoperative medication and hematological laboratory results, entity and location of primary cranial lesion, mode of anesthesia, time from skin closure to extubation, need for emergency head CT within 48 hours postoperatively, and indication for urgent return to the OR or ventriculostomy outside of the OR. Patients were followed for 30 days after the index surgery. The histology reports of all intracranial tumors were reviewed. The data was prospectively collected and anonymously entered in an electronic database. To limit any sources of bias, usual standard medical and anesthesiological care as well as routine neurosurgical practice as it is performed at our department were not altered in any way for this observational clinical trial. A total of eight board certified neurosurgeons at our institution performed the elective surgeries.

### Neuroanesthesia management—early extubation in the operating room

Neuroanesthesia management was performed and supervised by one neuroanesthesiologist (VK) and one additional board certified anesthesiologist. Total intravenous anesthesia (TIVA) was induced and maintained with propofol, fentanyl, and remifentanil. Propofol 2% was administered as a target controlled infusion; the effect-site concentration (Alaris^®^ PK syringe pump, Schnider model) was titrated according to the clinical assessment of consciousness as well as processed frontal electroencephalogram (Bispectral Index; Philips BIS^®^ module; target 40–50). For intraoperative analgesia, fentanyl (boli, 1–2μg/kg for a total of 7–10μg/kg body weight (BWT)) and remifentanil (continuous infusion, 0.2–0.4μg/kg/min BWT) were given intravenously. Preoperative mannitol (20% g/kg BWT) was routinely administered for all craniotomy cases and intravenous dexamethasone (8 mg every 8 hours) was added for all tumor cases. A short-acting non-depolarizing relaxant (rocuronium bromide [Esmeron^®^; Merck Sharp & Dohme AG] 0.6 mg/kg BWT) was used for intubation and as continuous infusion (0.2–0.6 mg/kg/h) to yield 1–2 train-of-five twitches in all cases except when monitoring of motor evoked potentials was required. Towards the end of the procedure (when the bone flap was secured), rocuronium was discontinued and the rate of propofol was gradually reduced. TIVA was discontinued after removal of the Mayfield skull clamp. Extubation criteria were: return of pharyngeal and laryngeal reflexes, sufficient spontaneous breathing, and adequate motor response to vocal command. In all patients, a brief neurologic assessment (obeying commands such as eye opening, motor function) is routinely performed either before or immediately after extubation. Speech and visual functions are also assessed shortly after extubation. Patients who were unable to obey commands remained intubated. Postoperative analgesia was achieved with intravenous acetaminophen (paracetamol) and metamizol-sodium. If needed, additional intravenous boli of fentanyl were also given. Early extubation was defined as removal of the endotracheal tube within one hour after skin closure. If time to extubation was more than one hour after skin closure, it was defined as late extubation.

### Postoperative care

As part of routine postoperative care after elective intracranial surgery at our department, immediate weaning from sedation and endotracheal extubation in the OR is aimed for as soon as skin closure is complete. Postoperative observation and neurological monitoring of the extubated and awakened patients was ensured bedside by one intensive care nurse and two intensive care physicians (one resident physician and one board certified intensivist) under the supervision of a neurointensivist (WJZ) at a dedicated neurosurgical intermediate care unit. Patients with regular neurological status were transferred to the ward the next day. Routine head CT within the first 48 hours postoperatively to search for complications such as intracranial hemorrhage or brain swelling was not performed. A head CT was only ordered in agreement with the surgeon for unexpected neurological findings, which we defined as progressive somnolence, any occurrence of a new and unexpected neurological deficit, seizure, or unexpected prolonged time to wake up after weaning from sedation. Early postoperative magnetic resonance imaging within 72 hours after brain tumor surgery to determine the extent of resection, are part of our standard care.

### Statistical analysis

Quantitative data are shown as mean ± standard deviation (SD); percentage and confidence intervals were computed using the Clopper-Pearson method. For this observational study a power analysis for sample size calculation was not conducted. To compare patients in the two groups with or without early head CT with respect to time of extubation, or need for early postoperative surgical intervention, we used the Chi-Square (χ^2^) test or Fisher’s exact test. For risk stratification for comparison of two samples with or without presumed risk factors an unpaired Student’s two-tailed t-test was used. Statistical significance was declared for p-values <0.05. Statistical analysis was performed using IBM SPSS Statistics, Version 21.0.

## Results

Between November 2011 and March 2014 (29 months) a total of 2604 intracranial procedures were performed and patients were consecutively screened for eligibility. Four hundred ninety-two patients (270 women, 222 men, mean age 55.4 years [SD 14.5, range 20–89 years]) with primary elective craniotomies were included for final analysis (Fig 1). The mean ASA score was 2.34 (SD 0.63, range 1–4). Craniotomies were performed for 360 supratentorial and 132 infratentorial lesions. Table 1 gives an overview of the distribution of cases with regard to intracranial compartment and lesion entity.

**Table 1:** Overview of 492 elective craniotomies.

Location and typ of lesion	No.
**Supratentorial**	**360**
Tumors	299
meningiomas	116
HGG	88
metastasis	51
LGG	21
other primary tumors	23
Vascular	61
aneurysms	35
cavernomas	13
AVM	6
DAVF	5
other	2
**Infratentorial**	**132**
Tumors	107
vestibular schwannoma	38
metastasis	23
meningiomas	17
HGG	5
LGG	5
epidermoid	4
HBL	3
other primary tumors	12
Vascular	6
cavernomas	4
AVM	2
Functional / developmental	19
MVD	13
vestibular neurectomy	4
suboccipital decompression	2

Indication for elective craniotomy subdivided into location and type of cerebral lesion.

HGG = high grade glioma, LGG = low grade glioma, AVM = arteriovenous malformation, DAVF = dural arteriovenous fistula, HBL = hemangioblastoma, MVD = microvascular decompression.

### Primary endpoint

#### Return to the OR or ventriculostomy within 48 hours postoperatively

Eight patients (1.6%; 6 women, 2 men) required urgent surgical intervention within 48 hours of the index surgery based on clinical and radiological findings (Table 2). Mean age of patients with emergency neurosurgical intervention was 49.4 years (SD 5.93), with only one patient over age 60 years. The ASA score showed no statistical significant difference between patients requiring urgent postoperative surgical intervention and patients with uneventful postoperative course (mean ASA score 2.35 (SD 0.65) vs. 2.43 (SD 0.63), respectively, p = 0.53). Of the eight patients requiring urgent surgical intervention, three had undergone infratentorial and five supratentorial craniotomies. The site of the primary craniotomy (supra- versus infratentorial craniotomy) had no impact on the risk of urgent surgical intervention (5 out of 360 and 3 out of 132, respectively; p = 0.69, odds ratio 1.65, 95%CI [0.39, 7.01]; Fisher’s exact test). All patients who underwent urgent intervention had prior early emergency CT imaging due to unexpected neurological worsening. The complications comprised intracranial hemorrhages (ICH) in four cases (0.8%), brain swelling in three cases, and acute obstructive hydrocephalus in one case. The four cases with ICH required recraniotomy, and the latter four cases required placement of external ventricular drains.

**Table 2:** Cases with emergency CT and surgical intervention.

Location and type of lesion	Total cases	CT	% of all cases	Surgical intervention	% of all cases
**Supratentorial**	**360**	**32**	**8.9**	**5**	**1.4**
Tumors	299	25	8.4	4	1.3
Vascular	61	7	11.5	1	1.6
**Infratentorial**	**132**	**11**	**8.3**	**3**	**2.3**
Tumors	107	10	9.3	3	2.8
Vascular	6	1	16.7	0	0
Functional, MVD, developmental	19	0	0	0	0
**TOTAL**	**492**	**43**	**8.7**	**8**	**1.6**

Overview of patients subdivided into location and type of cerebral lesion requiring emergency CT scanning and surgical intervention.

### Secondary endpoints

#### Duration of surgery

Mean duration of elective surgery (from skin incision to skin closure) was 271 minutes (SD 114) in the early extubation group and 289 minutes (SD 127) in the late extubation group (p = 0.47).

#### Time to extubation

Four hundred sixty-nine patients (95.3%) were extubated within one hour (early extubation group). Twenty-three (4.7%) patients were extubated with a delay of more than one hour after skin-closure: 16 (3.3% of all patients) within two hours, 4 (0.8% of all patients) within three hours, and one patient (0.2%) each within 4, 6 and 10 hours after skin closure. Thirteen percent of patients with failed early extubation (3 out of 23) required urgent surgical intervention, compared with only 1.1% (5 out of 469) of successfully early extubated patients. This association was statistically significant (p = 0.004, odds ratio 13.92, 95%CI [3.11, 62.37]; Fisher’s exact test).

#### Incidence and timing of emergency head CT within 48 hours

Emergency head CT within 48 hours was performed in 43 patients (8.7% of all patients) due to unexpected neurological findings, including seven patients who could not be extubated within one hour after surgery (Tables 2 and 3). CT scans were performed within 2 hours for six patients (14% of patients with emergency CT), between 2 and 6 hours for seven patients (16%) and between 6 and 48 hours after surgery for 30 patients (70%). In the early extubation group the emergency head CT rate within 48 hours after surgery was 7.7% (36 of 469) compared to 30.4% (7 of 23) in the delayed extubation group (p = 0.002, odds ratio 5.26, 95%CI [2.03, 13.62]; Fisher’s exact test) (Table 3).

**Table 3:** Association of extubation and emergency CT (< 48h).

	no CT	CT	total	rate
extubation > 1 h	16	7	23	30.4%
extubation < 1 h	433	36	469	7.7%
total	449	43	492	8.7%

*P* = 0.002, odds ratio 5.26 (95%CI 2.03–13.62); Fisher’s exact test.

Based on a yield of 8 out of 43 scanned patients (out of 492 included patients) requiring urgent surgical intervention sensitivity and specificity for ordering a head CT study with regard to urgent surgical intervention was 100% and 92.77%, respectively. The positive predictive value for surgical intervention was 18.6%.

#### Site of craniotomy, time to extubation and emergency head CT within 48 hours

Early extubation failed in 4.5% (n = 6) of patients after infratentorial craniotomy and in 4.7% (n = 17) of patients after a supratentorial approach. There was no statistically significant association between site of craniotomy (infra- vs. supratentorial) and failure of early extubation (p = 0.92, odds ratio 0.96, 95%CI [0.37, 2.49]; Chi-square test). Infratentorial lesions showed no increased risk for need of emergency head CT scanning compared to supratentorial lesions: CT scanning for infratentorial craniotomies was performed in 11 cases (8.3% of all infratentorial cases) and for 32 cases (8.9%) located supratentorially (Table 2) (p = 0.84, odds ratio 0.93, 95%CI [0.46, 1.91]; Chi-square test).

#### Mortality

The overall surgical mortality, defined as death within 30 days after the index surgery, was 0.2% (1 patient). This 27-year old male patient had undergone surgical debulking of a deep-seeded supratentorial glioblastoma and died 13 days after surgery.

## Discussion

The present study emphasizes the fact that routine head CT scanning early after elective craniotomy for a patient without unexpected neurological deterioration is unnecessary. We were able to adhere to this strategy in 449 cases (91.3%) with these patients having an uneventful postoperative course. Our study as well as previous and recent reports dealing with the utility of repeat head CT have demonstrated that this effort is feasible and compatible with patient safety [5–7].

An accurate definition of postoperative intracranial hemorrhage remains unclear and the range of reported rates in the literature varies substantially (from 0.8% to 50%) [8]. In the current consecutive series of 492 electively performed craniotomies the rate for emergency neurosurgical intervention within 48 hours was 1.6% with only half of these patients (0.8%) requiring recraniotomy for postoperative hemorrhage. These figures are comparable to published data regarding incidence of postoperative hemorrhage (0.8–2.1%) and recraniotomy (0.7–2.1%) [9–12]. Reported mortality in these studies range from 0.2–2.3% compared to 0.2% in our series. The mode of anesthesiological recovery and extubation time in these neurosurgical studies were not described.

In the present study we focused on one aspect of a “fast track paradigm”, namely early extubation after elective craniotomy. This approach of early extubation combined with close neurological surveillance requires a dedicated interdisciplinary team with the neurosurgeon, the neuroanesthesiologist and the neurointensivist as key players. Well-trained staff from all disciplines is mandatory. Early extubation immediately after elective cranial procedures may expose the patient to potential risks such as respiratory, metabolic and hemodynamic changes, pain, nausea and vomiting [13–16] and might increase the rate of seizures. However, our data show that early endotracheal extubation of patients after non-emergency craniotomy is feasible, safe, and does not increase perioperative morbidity or mortality. The safety and feasibility of early extubation after infratentorial craniotomy has been previously reported [17]. Postoperatively extubated and responsive patients can be more closely monitored at a highly specialized intermediate care unit or ICU. Successful early extubation in the OR has been shown to be highly predictive for an uneventful postoperative period [18], which is confirmed by our results. In our series, patients with failed early extubation had a significantly increased risk of requiring urgent surgical intervention within 48 hours as compared to patients with successful early extubation. With patients managed in a delayed recovery fashion with prolonged sedation and mechanical ventilation, early signs of intracranial mass effect such as depressed level of consciousness or focal neurological deficits could be missed and urgent intervention would be compromised. In such settings early postoperative CT scanning is back on the table.

Neuroanesthesia management was guided by standards that had been established in our institution. Care was provided by regular anesthesia personnel, including senior anesthesiologists, residents and nurses who were not aware of the study. Total intravenous anesthesia with propofol, fentanyl and remifentanil is widely used in neurosurgical procedures, but successful early extubation has also been reported with volatile anesthetics [19]. We prefer TIVA to volatile anesthesia because of a lower incidence of postoperative nausea and vomiting, and less interference with intraoperative monitoring of motor evoked potentials. But it is possible that similar results would be achieved with other anesthetic regimens, provided that neurologic assessment can be performed within the first hour after completion of surgery.

### One hour threshold—patient population that might profit from head CT

As stated earlier as a requirement for endotracheal extubation, the patient needs to pass a short neurological assessment performed by the anesthesiologist, which requires repeatedly obeying commands such as eye opening and squeezing a hand. Failure to extubate the patient within one hour after skin closure after uneventful elective cranial surgery should raise suspicion of possible early complications such as intracranial bleeding or edema formation. In accordance with our data this should be interpreted as an unexpected neurological finding warranting early postoperative CT. We propose that this one-hour rule should be implemented as a threshold for ordering an urgent head CT in neurosurgical patients.

The decision for urgent surgical intervention in our series was primarily based on the neurological examination that led to CT scanning. In the absence of unexpected neurological findings in alert and clinically monitored patients, we believe that early routine CT scans are unnecessary. This is in agreement with a recent retrospective study [4] as well as with a previous study that showed no impact of routine early postoperative CT on clinical management of patients without neurological decline in skull base surgery [20]. Furthermore, one has to keep in mind that transfer of an intubated patient to the imaging unit can cause additional harm, and reduces staff resources. Lastly, limiting unnecessary head CTs is certain to reduce health care costs as well as exposure of patients to unnecessary ionizing radiation with its risk of cancer development and other genetic damage [21, 22].

Whilst postoperative neurological deterioration poses a clear indication for urgent head CT scanning there are of course also surgical reasons for a scan after craniotomy, e.g. for confirmation of correct placement of electrodes and catheters after deep brain stimulation and ventriculoperitoneal shunting, respectively. However, we did not include these procedures in our analysis. Early postoperative MRI (within 72 hours) after glioma surgery was routinely done as it has become standard practice to evaluate the extent of resection [23]. If an unintended tumor remnant was seen re-do surgery to achieve complete resection of enhancing tumor was offered to patients a few days after the index craniotomy at our institution [24]. However, we intentionally focused only on head CT scanning and did not perform a systematic analysis of the impact of early postoperative MRIs for tumor patients in regard to clinically significant postoperative complications requiring emergent surgical intervention.

### Study limitations

Our conclusions may be limited to highly specialized neurosurgical centers with specially trained staff and established treatment algorithms. The presented study did not consider the potential confounding effect of specific comorbidities such as chronic pulmonary disease, cardiovascular disease or other chronic illnesses on time to extubation. We considered that the overall general physical health status and perioperative risk was reflected by the ASA score. Since we excluded awake craniotomies as well as pediatric and trauma patients, there might be a selection bias and our findings cannot be generalized to broader neurosurgical cohorts. Whether early extubation after elective craniotomy is safe or even superior to another regime can only be answered definitively with a randomized controlled trial. Further studies, preferably multicenter randomized trials with participating departments practicing one or the other postoperative patient management strategy (early or late extubation) are needed.

## Conclusions

Early extubation of patients in the OR after elective craniotomy procedures is safe and does not increase the rate of urgent surgical interventions or patient mortality. With this regime, routine early postoperative head CT scanning for detection of postoperative complications in conjunction with a normal neurological examination is not justified. CT scanning in the early postoperative period should be reserved for patients with unexpected neurological findings. Failure to extubate within one hour after skin closure should be considered “unexpected” and is predictive for postoperative complications leading to urgent neurosurgical intervention. Therefore unintended, delayed extubation (>1 hour) should serve as an indicator for obtaining an urgent head CT.

## Supporting Information

S1 File Study Protocol. (DOCX)

S2 File TREND checklist. (PDF)
