# Supplementary material for: No Routine Postoperative Head CT following Elective Craniotomy – A Paradigm Shift?
Source: PLoS One. 2016 Apr 14;11(4):e0153499. doi: 10.1371/journal.pone.0153499 (PMC4831779; doi:10.1371/journal.pone.0153499)
Supplement: S1 File — (DOCX) [file pone.0153499.s001.docx]

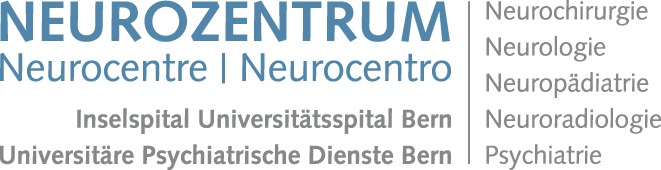


**Universitätsklinik für Neurochirurgie**

Direktor und Chefarzt

Prof. Dr. med. Andreas Raabe

www.neurochirurgie.insel.ch

Clinical Study Protocol

*Is fast-track management after elective cranial surgery safe and can it replace routine early postoperative computed tomography?*

(AGEK 1; SPIRIT #1)

Early extubation

| Study Type: | Beobachtungsstudie |
| --- | --- |
| Study Categorisation: | Kategorie A |
| Study Registration: | Clinical Trials.gov |
| Study Identifier: |  |
| Sponsor, Sponsor-Investigator or Principal Investigator: | Prof. Dr. med. Jürgen Beck |
| Investigational Product: |  |
| Protocol Version and Date: | Version 2.1 (20.10.2014) |

CONFIDENTIAL

e.g. “The information contained in this document is confidential and the property of the Universitätsklinik für Neurochirurgie (or “sponsor”). The information may not - in full or in part - be transmitted, reproduced, published, or disclosed to others than the applicable Competent Ethics Committee(s) and Regulatory Authority(ies) without prior written authorisation from the sponsor except to the extent necessary to obtain informed consent from those who will participate in the study.

Signature Page(s)

(AGEK 1.1; ICH E6 6.1)

ICH E6: Have signature pages with name and title of the person(s) authorised to sign the protocol and the protocol amendment(s) for the sponsor or of the medical expert (if applicable), the investigator responsible for conducting the trial, the statistician (if applicable)

Note: Add more lines, functions and pages if relevant, e.g for trial statistician, if relevant or protocol contributors

| Study number | NCT01987648 |
| --- | --- |
| Study Title | Is fast-track management after elective cranial surgery safe and can it replace routine early postoperative computed tomography? |

The Sponsor-Investigator and trial statistician have approved the protocol version 2 (24.06.2014) and confirm hereby to conduct the study according to the protocol, current version of the World Medical Association Declaration of Helsinki and the local legally applicable requirements.

Sponsor-Investigator:

Printed name of Sponsor-Investigator (if Sponsor and PI is not the same person please add an additional signature line for the PI of the study)

| Site | Universitätsklinik für Neurochirurgie, Inselspital Bern |
| --- | --- |
| Sponsor | Prof. Dr. med. Jürgen Beck |

| Place/Date |  | Signature |
| --- | --- | --- |

| Site | Universitätsklinik für Neurochirurgie, Inselspital Bern |
| --- | --- |
| Principal Investigator | Dr. med. Ralph Schär |

| Place/Date |  | Signature |
| --- | --- | --- |

Local Principal Investigator at study site*:

I have read and understood this trial protocol and agree to conduct the trial as set out in this study protocol, the current version of the World Medical Association Declaration of Helsinki, ICH-GCP guidelines or ISO 14155 norm and the local legally applicable requirements.

| Site | Universitätsklinik für Neurochirurgie, Inselspital Bern |
| --- | --- |
| Principal investigator | Dr. med. Ralph Schär |

| Place/Date |  | Signature |
| --- | --- | --- |

**Note:* In multicentric studies, this page must be individually signed by all participating Local Principal Investigators.

Table of Contents

Study synopsis 7

study summary in local language can be provided here 10

Abbreviations 11

Study schedule 12

1. STUDY ADMINISTRATIVE STRUCTURE 13

1.1 Sponsor, Sponsor-Investigator 13

1.2 Principal Investigator(s) 13

1.3 Statistician ("Biostatistician") 13

1.4 Laboratory 13

1.5 Monitoring institution 13

1.6 Data Safety Monitoring Committee 14

1.7 Any other relevant Committee, Person, Organisation, Institution 14

2. ETHICAL AND REGULATORY ASPECTS 14

2.1 Study registration 14

2.2 Categorisation of study (revise text once English translation is provided by BAG!) 14

2.3 Competent Ethics Committee (CEC) 14

2.4 Competent Authorities (CA) 15

2.5 Ethical Conduct of the Study 15

2.6 Declaration of interest 15

2.7 Patient Information and Informed Consent 15

2.8 Participant privacy and confidentiality 15

2.9 Early termination of the study 15

2.10 Protocol amendments 16

3. Background and Rationale 16

3.1 Background and Rationale 16

3.2 Investigational Product (treatment, device) and Indication 17

3.3 Preclinical Evidence 17

3.4 Clinical Evidence to Date 17

3.5 Dose Rationale / Medical Device: Rationale for the intended purpose in study (pre-market MD) 17

3.6 Explanation for choice of comparator (or placebo) 17

3.7 Risks / Benefits 17

3.8 Justification of choice of study population 17

4. STUDY OBJECTIVES 18

4.1 Overall Objective 18

4.2 Primary Objective 18

4.3 Secondary Objectives 18

4.4 Safety Objectives 18

5. STUDY OUTCOMES 19

5.1 Primary Outcome 19

5.2 Secondary Outcomes 19

5.3 Other Outcomes of Interest 19

5.4 Safety Outcomes 19

6. STUDY DESIGN 20

6.1 General study design and justification of design 20

6.2 Methods of minimising bias 20

6.2.1 Randomisation 20

6.2.2 Blinding procedures 20

6.2.3 Other methods of minimising bias 20

6.3 Unblinding Procedures (Code break) 20

7. STUDY POPULATION 21

7.1 Eligibility criteria 21

7.2 Recruitment and screening 21

7.3 Assignment to study groups 21

7.4 Criteria for withdrawal / discontinuation of participants 21

8. STUDY INTERVENTION 22

8.1 Identity of Investigational Products (treatment / medical device) 22

8.1.1 Experimental Intervention (treatment / medical device) 22

8.1.2 Control Intervention (standard/routine/comparator treatment / medical device) 22

8.1.3 Packaging, Labelling and Supply (re-supply) 22

8.1.4 Storage Conditions 22

8.2 Administration of experimental and control interventions 22

8.2.1 Experimental Intervention 22

8.2.2 Control Intervention 22

8.3 Dose / Device modifications 22

8.4 Compliance with study intervention 22

8.5 Data Collection and Follow-up for withdrawn participants 23

8.6 Trial specific preventive measures 23

8.7 Concomitant Interventions (treatments) 23

8.8 Study Drug / Medical Device Accountability 23

8.9 Return or Destruction of Study Drug / Medical Device 23

9. STUDY ASSESSMENTS 24

9.1 Study flow chart(s) / table of study procedures and assessments 24

9.2 Assessments of outcomes 24

9.2.1 Assessment of primary outcome 24

9.2.2 Assessment of secondary outcomes 24

9.2.3 Assessment of other outcomes of interest 24

9.2.4 Assessment of safety outcomes 24

9.2.5 Assessments in participants who prematurely stop the study 25

9.3 Procedures at each visit 25

9.3.1 Split into subtitles by type of visit 25

9.3.2 Split into subtitles by type of visit 25

9.3.3 Split into subtitles by type of visit 25

10. SAFETY 26

10.1 Drug studies 26

10.1.1 Definition and assessment of (serious) adverse events and other safety related events 26

10.1.2 Reporting of serious adverse events (SAE) and other safety related events 26

10.1.3 Follow up of (Serious) Adverse Events 26

10.2 Medical Device Category C studies 26

10.2.1 Definition and Assessment of (Serious) Adverse Events and other safety related events 26

10.2.2 Reporting of (Serious) Adverse Events and other safety related events 26

10.2.3 Follow up of (Serious) Adverse Events 26

10.3 Medical Device Category A studies 26

10.3.1 Definition and Assessment of safety related events 26

10.3.2 Reporting of Safety related events 26

11. STATISTICAL METHODS 27

11.1 Hypothesis 27

11.2 Determination of Sample Size 27

11.3 Statistical criteria of termination of trial 27

11.4 Planned Analyses 27

11.4.1 Datasets to be analysed, analysis populations 27

11.4.2 Primary Analysis 28

11.4.3 Secondary Analyses 28

11.4.4 Interim analyses 28

11.4.5 Safety analysis 28

11.4.6 Deviation(s) from the original statistical plan 28

11.5 Handling of missing data and drop-outs 28

12. QUALITY ASSURANCE AND CONTROL 29

12.1 Data handling and record keeping / archiving 29

12.1.1 Case Report Forms 29

12.1.2 Specification of source documents 29

12.1.3 Record keeping / archiving 29

12.2 Data management 29

12.2.1 Data Management System 29

12.2.2 Data security, access and back-up 29

12.2.3 Analysis and archiving 30

12.2.4 Electronic and central data validation 30

12.3 Monitoring 30

12.4 Audits and Inspections 30

12.5 Confidentiality, Data Protection 30

12.6 Storage of biological material and related health data 30

13. PUBLICATION AND DISSEMINATION POLICY 30

14. FUNDING AND SUPPORT 31

14.1 Funding 31

14.2 Other Support 31

15. INSURANCE 31

16. REFERENCES 32

17. APPENDICES 33

Study synopsis

(KlinV, Apendix 3, 1.1, 2.1, 3.1, 4.1; Appendix 5, 2b; AGEK Summary)

| Sponsor / Sponsor-Investigator | Prof. Dr. med. Jürgen Beck |
| --- | --- |
| Study Title: | Is fast-track management after elective cranial surgery safe and can it replace routine early postoperative computed tomography? |
| Short Title / Study ID: | Fast-track neurosurgery |
| Protocol Version and Date: | Version 2.1 (20.10.2014) |
| Trial registration: | Clinical Trials.gov (NCT01987648) |
| Study category and Rationale | Kategorie A |
| Clinical Phase: | For clinical trials with drugs: Clinical study phase or phase of clinical development (e.g. Phase 1, 2, 3 or 4; or according to ICH E8 para 3.1.3 Human Pharmacology, Therapeutic Exploratory, Therapeutic Confirmatory or Therapeutic Use); in case of Medical Device study rename and use e.g. “Phase of development” |
| Background and Rationale: | Postoperative patient management following elective cranial surgery may vary substantially between different neurosurgical institutions. However, the common objective in this crucial period is to avoid early postoperative complications (e.g. intracranial hemorrhaging or brain swelling). To detect these complications or rather to rule them out early routine cranial computed tomography used to be standard care and is still often performed despite the absence of unexpected neurological deficits. This practice is not substantiated by hard evidence.  There is no bedside monitoring to diagnose postoperative complications at a very early stage.  Depending on the neurosurgeon’s, anesthesist’s and neurointensivist’s policy and consensus one of the two following major strategies for postoperative care in cranial neurosurgery is preferred. On the one hand, in fear of hemodynamic and metabolic changes during emergence of anesthesia a delayed recovery is aimed for in order to strictly avoid periods of arterial hypertension, hypoventilation with CO_2_ increase, low oxygen saturation, extensive coughing, pressing, and pain. On the other hand early recovery and extubation is sought for monitoring of the awakened and responsive patient as early as possible for changes in level of consciousness or the development of unexpected neurological deficits. Evidently, these strategies are not compatible to one another. There is little evidence in the literature to suggest clear superiority of one regime over the other. |
| Objective(s): | We hypothesize that fast-track management of patients following elective craniotomy, that is early emergence from anaesthesia and extubation and transfer of the awakened and responsive patient either to a critical care or intermediate care unit is safe and does not increase the rate of return to OR compared to published data. Secondly, by continuous neurological and clinical monitoring we believe early routine cranial computed tomography for detection of postoperative hemorrhaging or swelling in the absence of unexpected neurological deterioration is not warranted. |
| Outcome(s): | **Primary Endpoint:**  Morbidity endpoint:  Incidence of early (<48 hrs) postoperative intracranial hemorrhaging or brain swelling warranting operative intervention/re-craniotomy and or death within 30 days of the index-surgery. **Secondary Endpoint:**  - 1. Event endpoint      - re-intubation      - postoperative epilepsy      - unscheduled emergency CT within 48 hrs after surgery      - return to OR      - emergency of anesthesia longer than 6 hrs after surgery   Length of hospitalization in intensiv care unit or intermediate care unit |
| Study design: | open label, no Randomisation |
| Inclusion / Exclusion criteria: | **Inclusion criteria:**   - planned elective craniotomy due to intracranical lesion - age older than 18 years   **Exclusion criteria:**   - biopsy only - re-craniotomy or other operative revision - craniotomy due to infection - awake surgery |
| Measurements and procedures: | The university clinic of neurosurgery in Bern will prospectively collect data of patients who meet the inclusion/exclusion criteria. The data will be anonymously entered in a database.  Routine standard of care will not be altered in any way. No study-specific interventions, medical devices or products will or are being used or tested for this clinical study. |
| Study Product / Intervention: | None |
| Control Intervention (if applicable): | - |
| Number of Participants with Rationale: | 1000 in accordance with published patient collectives in other observational studies with similar objectives in clinical research in neurosurgery |
| Study Duration: | 11/2011 - 06/2016 (31 months) |
| Study Schedule: | First-Participant-In: 11/2011  Last-Participant-Out: 06/2016 |
| Investigator(s): | Prof. Dr. med. Jürgen Beck  Universitätsklinik für Neurochirurgie  Email: [juergen.beck@insel.ch](mailto:juergen.beck@insel.ch)  Tel.: 031 632 2409  Dr. med. Ralph Schär  Universitätsklinik für Neurochirurgie  Email: ralph.schaer@insel.ch  Tel.: 031 632 2409  Prof. Dr. med. Andreas Raabe  Universitätsklinik für Neurochirurgie  Email: andreas.raabe@insel.ch  Tel.: 031 632 2409  Dr. med. Michael Fiechter  Universitätsklinik für Neurochirurgie  Email: michael.fiechter@insel.ch  Tel.: 031 632 2409 |
| Study Centre(s): | Single-centre |
| Statistical Considerations: | First analysis of 500 patients, second analysis of 1000 patients. Event rate will be calculated in percent. The exact Fisher test for analysis of significance of correlation with timing of extubation and need for unscheduled early CT examination of examined events will be used.  Comparison of our event-rates with published data in the literature will also be evaluated. |
| GCP Statement: | This study will be conducted in compliance with the protocol, the current version of the Declaration of Helsinki as well as all national legal and regulatory requirements. |

study summary in local language can be provided here

#### **Hypothese:**

Wir sind der Meinung, dass die unverzügliche Extubation, sofern dies aus anästhesiologischer und neurochirurgischer Sicht vertretbar ist, und fortwährende klinisch-neurologische Überwachung von elektiv kraniotomierten Patienten nicht mit einer höheren Komplikationsrate einhergeht, verglichen mit derjenigen Strategie, bei der dieses Patientenkollektiv postoperativ auf der Intensivstation mehrere Stunden intubiert und sediert bleibt. Im Gegenteil, durch die engmaschige Überwachung wacher Patienten kann eine klinisch-neurologische Verschlechterung rascher erkannt und entsprechende Massnahmen ergriffen werden. Zudem scheint eine früh-postoperative routine-mässige Schädel-CT-Untersuchung ohne unerwartete neurologische Verschlechterung nicht gerechtfertigt.

#### **Ziel dieser Studie:**

Bis heute existiert in der Literatur keine klare Evidenz über das postoperative Management nach elektiven Kraniotomien hinsichtlich des Zeitpunktes für Sedationsstopp und Extubation. Unser Ziel ist es, aufzuzeigen, dass das rasche Extubieren nach elektiven Kraniotomien für die Patienten hinsichtlich der Komplikationsrate keinen nachteiligen Effekt nach sich zieht und dass dadurch auf eine Routine-CT-Untersuchung des Schädels zum Ausschluss von postoperativen Komplikationen verzichtet werden kann.

Abbreviations

Provide a list of abbreviations used on the protocol - to be completed

| AE | Adverse Event |
| --- | --- |
| CA | Competent Authority (e.g. Swissmedic) |
| CEC | Competent Ethics Committee |
| CRF | Case Report Form |
| eCRF | Electronic Case Report Form |
| CTCAE | Common terminology criteria for adverse events |
| DSUR | Development safety update report |
| GCP | Good Clinical Practice |
| IB | Investigator’s Brochure |
| Ho | Null hypothesis |
| H1 | Alternative hypothesis |
| HFG | Humanforschungsgesetz (Law on human research) |
| HMG | Heilmittelgesetz |
| IMP | Investigational Medicinal Product |
| IIT | Investigator-initiated Trial |
| ISO | International Organisation for Standardisation |
| ITT | Intention to treat |
| KlinV | Verordnung über klinische Versuche in der Humanforschung |
| LHR | Law on human research |
| LPTh | Loi sur les produits thérapeutiques |
| LRH | Loi fédérale relative à la recherche sur l’être humain |
| MD | Medical Device |
| OClin | Ordonnance sur les essais cliniques dans le cadre de la recherche sur l'être humain |
| PI | Principal Investigator |
| SDV | Source Data Verification |
| SOP | Standard Operating Procedure |
| SPC | Summary of product characteristics |
| SUSAR | Suspected Unexpected Serious Adverse Reaction |
| TMF | Trial Master File |

Study schedule

(AGEK 4.2; SPIRIT #13; ICH E6 6.4.2)

Insert a flow chart (graphic) or tabular listing of schedule of events and assessments and procedures of the study (an example is provided below, amend and expand according to the specific study). To be repeated in 9.1.

e.g.:

| Study Periods | Screening | Treatment, Intervention Period | Life Status |
| --- | --- | --- | --- |
| Visit | 1 | 1 | 2 |
| Time (hour, day, week) |  | 48 Hours after Surgey | 30 Days after Surgery |
| Demographics | x |  |  |
| Medical History | x |  |  |
| In- /Exclusion Criteria | x |  |  |
| Physical Examination | x | x |  |
| Laboratory Tests | x |  |  |
| Primary Variables | x | x |  |
| Secondary Variables | x | x |  |
| Adverse Events |  | X |  |
| Life Status |  |  | x |

# STUDY ADMINISTRATIVE STRUCTURE

(ICH/E6 6.1.2-6.1.7; AGEK 1.1; SPIRIT 5a-d)

## Sponsor, Sponsor-Investigator

(ICH/E6 6.1.2; AGEK 1.1; SPIRIT 5b)

ICH: Name and address of the sponsor …..

Prof. Dr. med. Jürgen Beck

Universitätsklinik für Neurochirurgie, Inselspital Bern

Email: [juergen.beck@insel.ch](mailto:juergen.beck@insel.ch)

Telefon: 031 632 22409

## Principal Investigator(s)

(ICH/E6 6.1.5, 6.1.6; AGEK 1.1; SPIRIT 5a-d)

ICH: Name and title of the investigator(s) who is (are) responsible for conducting the trial, and the address and telephone number(s) of the trial site(s).

Dr. med. Ralph Schär

Universitätsklinik für Neurochirurgie

Email: ralph.schaer@insel.ch

Tel.: 031 632 22409

Prof. Dr. med. Andreas Raabe

Universitätsklinik für Neurochirurgie

Email: andreas.raabe@insel.ch

Tel.: 031 632 22409

Dr. med. Michael Fiechter

Universitätsklinik für Neurochirurgie

Email: michael.fiechter@insel.ch

Tel.: 031 632 22409

## Statistician ("Biostatistician")

(ICH/E6 6.1.7; SPIRIT 5a-d)

ICH: Name(s) and address(es) of the clinical laboratory(ies) and other medical and/or technical department(s) and/or institutions involved in the trial.

Dr. med. Et sc. Nat. Corrado Bernasconi

Universitätsklinik für Neurologie

Email: corrado.bernasconi@insel.ch

Tel.: 031 632 3140

## Laboratory

(ICH/E6 6.1.7; SPIRIT 5a-d)

ICH: Name(s) and address(es) of the clinical laboratory(ies) …………. involved in the trial.

Not applicable

## Monitoring institution

(ICH/E6 6.1.2; SPIRIT 5a-d)

ICH: Name and address of the …. monitor (if other than the sponsor).

Not applicable

## Data Safety Monitoring Committee

(ICH/E6 6.1.7; SPIRIT 5a-d)

ICH: Name(s) and address(es) of the clinical laboratory(ies) and other medical and/or technical department(s) and/or institutions involved in the trial.

Not applicable

## Any other relevant Committee, Person, Organisation, Institution

(ICH/E6 6.1.7; SPIRIT 5a-d)

ICH: Name(s) and address(es) of the clinical laboratory(ies) and other medical and/or technical department(s) and/or institutions involved in the trial.

Nicole Söll

Universitätsklinik für Neurochirurgie

Email: nicole.soell@insel.ch

Tel.: 031 632 23164

Study coordination and data management

# ETHICAL AND REGULATORY ASPECTS

(ICH/E6 6.12; AGEK 11; SPIRIT #24, 5)

ICH: Description of ethical considerations relating to the trial.

The decision of the CEC and Swissmedic/foreign competent authority concerning the conduct of the study will be made in writing to the Sponsor-Investigator before commencement of this study. The clinical study can only begin once approval from all required authorities has been received. Any additional requirements imposed by the authorities shall be implemented.

## Study registration

(KlinV, Art. 1d, 64; SPIRIT #2a-b)

The study will be registered in a registry listed in the WHO International Clinical Trials Registry Platform and in a national language in the Swiss Federal Complementary Database.

## Categorisation of study (revise text once English translation is provided by BAG!)

(KlinV, Art. 19, 20, App 3, 1.1)

Category A.

## Competent Ethics Committee (CEC)

(KlinV, Art 24-29; SPIRIT #24)

The responsible investigator ensures that approval of an appropriately constituted Competent Ethics Committee (CEC) is sought for the clinical study.

No changes are made to the protocol without prior Sponsor and CEC approval.

Premature study end or interruption of the study is reported within 15 days. The regular end of the study is reported to the CEC within 90 days, the final study report shall be submitted within one year after study end. Amendments are reported according to chapter 3.10.

## Competent Authorities (CA)

(KlinV, Art. 23, 27, 30-39, 42, 43, 46-48, 57; SPIRIT #24)

Not applicable

## Ethical Conduct of the Study

(KlinV, Art. 5; AGEK 11; ICH E6 6.12, 6.2.5)

ICH: A statement that the trial will be conducted in compliance with the protocol, GCP and the applicable regulatory requirement(s).

The study will be carried out in accordance to the protocol and with principles enunciated in the current version of the Declaration of Helsinki, the guidelines of Good Clinical Practice (GCP) issued by ICH, in case of medical device: the European Directive on medical devices 93/42/EEC and the ISO Norm 14155 and ISO 14971, the Swiss Law and Swiss regulatory authority’s requirements. The CEC and regulatory authorities will receive annual safety and interim reports and be informed about study stop/end in agreement with local requirements.

## Declaration of interest

(KlinV, Art. 3b; SPIRIT #28)

No conflict of interest.

## Patient Information and Informed Consent

(KlinV, Art. 7-9, Art. 15-17, Appendix 3, 1.4, 2.4, 3.4, 4.3, Appendix 4, 3.6; AGEK submission checklist item 5; SPIRIT #26, 32)

There will be no consent from the patient for the collection of their data. Collecting an informed consent from every patient is disproportional.

## Participant privacy and confidentiality

(KlinV, Art. 18; ICH/E6 6.10; AGEK 12.2, SPIRIT #27)

ICH: The sponsor should ensure that it is specified in the protocol or other written agreement that the investigator(s)/institution(s) will permit trial-related monitoring, audits, IRB/IEC review, and regulatory inspection(s), providing direct access to source data/documents.

The investigator affirms and upholds the principle of the participant's right to privacy and that they shall comply with applicable privacy laws. Especially, anonymity of the participants will be guaranteed when presenting the data at scientific meetings or publishing them in scientific journals.

Individual subject medical information obtained as a result of this study is considered confidential and disclosure to third parties is prohibited. Subject confidentiality will be further ensured by utilising subject identification code numbers to correspond to treatment data in the computer files.

For data verification purposes, authorised representatives of the Sponsor (-Investigator), a competent authority (e.g. Swissmedic), or an ethics committee may require direct access to parts of the medical records relevant to the study, including participants’ medical history.

## Early termination of the study

(KlinV Art. 47; ICH/E6 6.4.6; SPIRIT #21b)

ICH: A description of the "stopping rules" or "discontinuation criteria" for individual participants, parts of trial and entire trial.

The Sponsor-Investigator may terminate the study prematurely according to certain circumstances, for example:

- ethical concerns,
- insufficient participant recruitment,
- alterations in accepted clinical practice that make the continuation of a clinical trial unwise,
- early evidence of benefit or harm

## Protocol amendments

(KlinV, Art. 29, 34, 55; SPIRIT #25)

Prof. J. Beck and Dr. R. Schär are allowed the make amendments to the protocol.

# Background and Rationale

(ICH 6.2; AGEK 3; SPIRIT #6)

## Background and Rationale

(ICH/E6 6.2; AGEK 3.1; SPIRIT #6)

Postoperative patient management following elective cranial surgery may vary substantially between different neurosurgical institutions, depending on the neurosurgeons preference or policy and neuroanesthesiological or neurointensivists’ resources for the most part. However, the common objective in this crucial period is to avoid any early postoperative complications such as intracranial hemorrhaging or brain swelling. To detect these feared complications after cranial surgery or rather to rule them out early routine cranial computed tomography within the first hours used to be standard care^[[1]](#endnote-1)^ and is still often performed despite the absence of unexpected neurological deficits. This practice is not substantiated by hard evidence and scans are mainly ordered by recommendation and training backroud of the neurosurgeon.

Unfortunately, there is no bedside monitoring to diagnose postoperative complications at a very early stage. Patients become symptomatic by a neurological deterioration such as an altered level of consciousness or an early postoperative epileptic seizure. Therefore, much attention has been paid to provide a close monitoring and observation of the patient usually at a critical care unit during the first hours after brain surgery.

Depending on the neurosurgeon’s, anesthesist’s and neurointensivist’s policy and consensus one of the two following major strategies for postoperative care in cranial neurosurgery is preferred. On the one hand, in fear of hemodynamic and metabolic changes due to sympathetic overdrive during emergence of anesthesia a delayed recovery is aimed for in order to strictly avoid periods of arterial hypertension, hypoventilation with CO_2_ increase, low oxygen saturation, extensive coughing, pressing, and pain. On the other hand early recovery and extubation is sought for monitoring of the awakened and responsive patient as early as possible for changes in level of consciousness or the development of unexpected neurological deficits. Evidently, these strategies are not compatible to one another. The first strategy requires keeping the patient sedated and intubated over a longer period of time and weaning from sedation and mechanical ventilation is performed later on at the critical care unit. This hinders early detection of an unexpected neurological deterioration and timely reaction. On the contrary, the second fast-track strategy aims at an early recovery and extubation of the patient immediately after completing the surgical procedure. On the downside this may expose the patient to more hemodynamic and metabolical stress possibly increasing the risk of early postoperative complications. There is little evidence in the literature to suggest clear superiority of one regime over the other.

We hypothesize that fast-track management of patients following elective craniotomy, that is early emergence from anaesthesia and extubation and transfer of the awakened and responsive patient either to a critical care or intermediate care unit is safe and does not increase the rate of return to OR compared to published data. Secondly, by continuous neurological and clinical monitoring we believe early routine cranial computed tomography for detection of postoperative hemorrhaging or swelling in the absence of unexpected neurological deterioration is not warranted.

## Investigational Product (treatment, device) and Indication

(ICH/E6 6.2.1; AGEK 2; SPIRIT #6)

ICH: Name and description of the investigational product(s).

Not applicable

## Preclinical Evidence

(ICH/E6 6.2.2; SPIRIT #6a)

ICH: A summary of findings from nonclinical studies that potentially have clinical significance …..

Not applicable

## Clinical Evidence to Date

(ICH/E6 6.2.2; SPIRIT #6a)

ICH: A summary of findings from … and from clinical trials that are relevant to the trial.

An accurate definition of postoperative intracranial hemorrhage remains unclear and the range of reported rates in the literature is substantial (from 0.8% to 50%). Lassen et al. reported a surgical mortality of 2.3% at 30 days in their series of 2630 consecutive craniotomies for intracranial tumors and postoperative hematomas requiring recraniotomy occurred in 2.1% of all cases. In another large series of 2305 cases 50 postoperative hematomas were detected (2.2%) with 24 patients requiring emergent operative intervention (1%). Overall mortality was 0.4% (9 patients). In an even larger series of nearly 5000 consecutive intracranial procedures postoperative hemorrhage was noted in a mere 0.8% of all cases. Unfortunately, the mode of anesthesiological recovery and extubation time in these neurosurgical studies are not described.

## Dose Rationale / Medical Device: Rationale for the intended purpose in study (pre-market MD)

(ICH/E6 6.2.4; SPIRIT #6a)

ICH: Description of and justification for the route of administration, dosage, dosage regimen, and treatment period(s).

Not applicable

## Explanation for choice of comparator (or placebo)

(AGEK 11.3; SPIRIT #6b)

Not applicable

## Risks / Benefits

(KlinV, Appendix 4, 3.5; Art 25d2; ICH/E6 6.2.3; AGEK 11.1; SPIRIT #6a; MD: ISO 14155 Annex A & ISO 14971)

ICH: Summary of the known and potential risks and benefits, if any, to human subjects.

This is an observational study, no study-specific interventions will be performed, no study-specific medical devices or drugs will be tested. Therefore there are no additional risks or benefits for patients.

## Justification of choice of study population

(KlinV, Art 25d4, Art. 15-17; ICH/E6 6.2.6; AGEK 11.2)

ICH: Description of the population to be studied.

The sample size is set to be 1000 patients in accordance with published patient collectives in other observational studies with similar objectives in clinical research in neurosurgery.

# STUDY OBJECTIVES

(ICH/E6 6.3; AGEK 3; SPIRIT #7)

ICH: A detailed description of the objectives and the purpose of the trial.

## Overall Objective

We hypothesize that fast-track management of patients following elective craniotomy, that is early emergence from anaesthesia and extubation and transfer of the awakened and responsive patient either to a critical care or intermediate care unit is safe and does not increase the rate of return to OR compared to published data. Secondly, by continuous neurological and clinical monitoring we believe early routine cranial computed tomography for detection of postoperative hemorrhaging or swelling in the absence of unexpected neurological deterioration is not warranted.

## Primary Objective

To show that fast-track management after elective cranial surgery is safe.

## Secondary Objectives

To show that routine early postoperative CT after elective cranial surgery in awakened patients is unnecessary without evidence of unexpected neurological worsening.

## Safety Objectives

In studies with efficacy as primary and secondary endpoints safety is always an additional objective.

The study aims to assess the safety of fast-track management after elective cranial surgery.

# STUDY OUTCOMES

(ICH/E6 6.4.1; AGEK 4.1; SPIRIT #12)

ICH: A specific statement of the primary endpoints and the secondary endpoints, if any, to be measured during the trial.

## Primary Outcome

Morbidity endpoint:

Incidence of early (<48 hrs) postoperative intracranial hemorrhaging or brain swelling warranting operative intervention/re-craniotomy and or death within 30 days of the index-surgery.

## Secondary Outcomes

Event endpoint

- - - re-intubation
    - postoperative epilepsy
    - unscheduled emergency CT within 48 hrs after surgery
    - return to OR
    - emergency of anesthesia longer than 6 hrs after surgery

Length of hospitalization in intensiv care unit or intermediate care unit

## Other Outcomes of Interest

Not applicable

## Safety Outcomes

Not applicable since no planned study interventions, no study drug, no study devices

# STUDY DESIGN

(ICH/E6 6.4; AGEK 4; SPIRIT #8)

## General study design and justification of design

(ICH/E6 6.4.2, 6.4.5; AGEK 4.2; SPIRIT #8)

ICH: The scientific integrity of the trial and the credibility of the data from the trial depend substantially on the trial design.

ICH: A description of the type/design of trial to be conducted (e.g., double-blind, placebo-controlled, parallel design) and a schematic diagram of trial design, procedures and stages.

ICH: The expected duration of subject participation, and a description of the sequence and duration of all trial periods, including follow-up, if any.

Observational, prospective non-randomized single-center study

- Description of safety outcomes and comparison with historical data
- no study-specific interventions
- 1000 patients needing elective craniotomy due to intracranial lesions
- no blinding or masking
- no comparators
- no randomisation

## Methods of minimising bias

(ICH/E6 6.4.3; AGEK 4.3; SPIRIT #16, 17)

ICH: A description of the measures taken to minimize/avoid bias, including: Randomization, Blinding.

### Randomisation

Not applicable

### Blinding procedures

Not applicable

### Other methods of minimising bias

Patients admitted for elective craniotomy procedures at our department were consecutively screened and included if eligibility criteria were full field.

## Unblinding Procedures (Code break)

(ICH/E6 6.4.8; AGEK 4.2; SPIRIT #17b)

ICH: Maintenance of trial treatment randomization codes and procedures for breaking codes.

Not applicable

# STUDY POPULATION

(ICH/E6 6.2.6, 6.4.6; AGEK 3.2, 5; SPIRIT #9, 10, 15, 16, 21)

ICH: Description of the population to be studied.

## Eligibility criteria

(KlinV, Art 25d5; ICH/E6 6.5.1&6.5.2; AGEK 5.2&5.3; SPIRIT #10)

ICH: Subject inclusion and exclusion criteria.

All patients older than 18 years of age who are planned to have an elective craniotomy procedure at the university clinic of neurosurgery in Bern are eligible for the study.

**Inclusion criteria:**

- planned elective craniotomy due to intracranical lesion
- age older than 18 years

**Exclusion criteria:**

- biopsy only
- re-craniotomy or other operative revision
- craniotomy due to infection
- awake surgery

## Recruitment and screening

(KlinV, Art 25, Appendix 3, 1.4 & 1.6; AGEK 5.1; SPIRIT #15)

All elective craniotomy prodedures will be screened by our research nurse (N. Söll) for eligibility based on inclusion and exclusion criteria. There will be no payment or compensation for participants.

## Assignment to study groups

(AGEK 5; SPIRIT #16)

Not applicable

## Criteria for withdrawal / discontinuation of participants

(KlinV, Art 9; ICH/E6 6.5.3; SPIRIT #21b)

Subject withdrawal criteria (i.e., terminating investigational product treatment/trial treatment) and procedures specifying: a) When and how to withdraw subjects from the trial/ investigational product treatment. c) Whether and how subjects are to be replaced.

Patients will be informed by the study investigators or other medical doctor that their patient data will be collected. Patients can ask to be withdrawn at any time from the study and that their patient data shall not be collected.

# STUDY INTERVENTION

(SPIRIT #11)

## Identity of Investigational Products (treatment / medical device)

(ICH/E6 6.2.1, 6.4.2, 6.4.4; AGEK Checklist 2, item 3)

ICH: A description of the trial treatment(s) and the dosage and dosage regimen of the investigational product(s).

Not applicable. No interventions needed for this study.

### Experimental Intervention (treatment / medical device)

ICH: Name and description of the investigational product(s).

Not applicable

### Control Intervention (standard/routine/comparator treatment / medical device)

ICH: Name and description of the investigational product(s).

Not applicable

### Packaging, Labelling and Supply (re-supply)

ICH: Also include a description of the dosage form, packaging, and labelling of the investigational product(s).

Not applicable

### Storage Conditions

Not applicable

## Administration of experimental and control interventions

(ICH/E6 6.4.4)

### Experimental Intervention

ICH: Description of and justification of the treatment(s) to be administered, including the name(s) of all the product(s), the dose(s), the dosing schedule(s), the route/mode(s) of administration, and the treatment period(s), including the follow-up period(s) for subjects for each investigational product treatment/trial treatment group/arm of the trial.

Not applicable

### Control Intervention

ICH: Description of and justification of the treatment(s) to be administered, including the name(s) of all the product(s), the dose(s), the dosing schedule(s), the route/mode(s) of administration, and the treatment period(s), including the follow-up period(s) for subjects for each investigational product treatment/trial treatment group/arm of the trial.

Not applicable

## Dose / Device modifications

(SPIRIT #11b)

Not applicable

## Compliance with study intervention

(ICH/E6 6.6.3; AGEK Checklist 2, item 2; SPIRIT #11c)

ICH: Procedures for monitoring subject compliance.

Not applicable

## Data Collection and Follow-up for withdrawn participants

(ICH/E6 6.5.3; AGEK 9.2; SPIRIT #18b)

ICH: ………..b) The type and timing of the data to be collected for withdrawn subjects. d) The follow-up for subjects withdrawn from investigational product treatment/trial treatment.

The follow up period for this study is 30 days. There will be no study-specific follow up on patients who wish to be withdrawn or are withdrawn for any other reasons other than routine clinical follow up as ordered by the operating surgeon in accordance with standard medical care.

## Trial specific preventive measures

(ICH/E6 6.6.2; AGEK 9; SPIRIT #11d)

ICH: Medication(s)/treatment(s) permitted (including rescue medication) and not permitted before and/or during the trial.

Not applicable

## Concomitant Interventions (treatments)

(ICH/E6 6.6.2; AGEK 9; SPIRIT #11d)

ICH: Medication(s)/treatment(s) permitted (including rescue medication) and not permitted before and/or during the trial.

Not applicable

## Study Drug / Medical Device Accountability

(ICH/E6 6.4.7; AGEK Checklist 2, item 1; SPIRIT 11c)

ICH: Accountability procedures for the investigational product(s), including the placebo(s) and comparator(s), if any.

Not applicable

## Return or Destruction of Study Drug / Medical Device

(AGEK Checklist 2, item 1; SPIRIT 11c)

Not applicable

# STUDY ASSESSMENTS

(ICH/E6 6.7, 6.8; AGEK 6, 7; SPIRIT #18a)

Describe procedures, measurements, collection, storage of samples taken, etc.

## Study flow chart(s) / table of study procedures and assessments

30 Day

Life Status

Surgery

Postoperative Care: Complications, Procedures and Interventions within 48 Hours after surgery

## Assessments of outcomes

ICH: Specification of the efficacy parameters. Specification of safety parameters.

### Assessment of primary outcome

ICH: Methods and timing for assessing, recording, and analysing of efficacy & safety parameters.

The primary outcome will be assessed during his medical treatment by the treating medical physican and will be kept in the medical records. Mortality after 30 days of the elective craniotomy will be assessed by a study nurse.

### Assessment of secondary outcomes

ICH: Methods and timing for assessing, recording, and analysing of efficacy & safety parameters.

All secondary outcomes (48 hours after index surgery) will be assessed during his medical treatment by the treating medical physician and will be kept in the medical records.

### Assessment of other outcomes of interest

ICH: Methods and timing for assessing, recording, and analysing of efficacy & safety parameters.

Not applicable

### Assessment of safety outcomes

ICH E6 6.8: Specification of safety parameters. The methods and timing for assessing, recording, and analysing safety parameters

Not applicable

#### Adverse events

Not applicable

#### Laboratory parameters

For this observational study 2 laboratory parameter will be assessed: Quick (INR) and platelet count. Both parameters will be done preoperative and are standard procedure. No laboratory test will be done or ordered due to study.

#### Vital signs

Not applicable

### Assessments in participants who prematurely stop the study

There will be no study-specific assessments on patients who wish to be withdrawn or are withdrawn for any other reasons other than routine assessments as ordered by the operating surgeon in accordance with standard medical care.

## Procedures at each visit

Not applicable

### Split into subtitles by type of visit

### Split into subtitles by type of visit

### Split into subtitles by type of visit

# SAFETY

(KlinV Art. 37-43; ICH/E6 6.8; ISO14155 8.2.5, A.14; AGEK 4.1; SPIRIT # 22, 30)

## Drug studies

Not applicable

### Definition and assessment of (serious) adverse events and other safety related events

ICH: Procedures for eliciting reports of and for recording … adverse event and intercurrent illnesses.

### Reporting of serious adverse events (SAE) and other safety related events

(KlinV Art. 37)

ICH: Procedures for … reporting adverse event and intercurrent illnesses.

### Follow up of (Serious) Adverse Events

(ICH/E6 6.8.4; SPIRIT #30)

ICH: The type and duration of the follow-up of subjects after adverse events.

## Medical Device Category C studies

Not applicable

### Definition and Assessment of (Serious) Adverse Events and other safety related events

(MD: ISO 14155)

### Reporting of (Serious) Adverse Events and other safety related events

### Follow up of (Serious) Adverse Events

(SPIRIT #30)

## Medical Device Category A studies

Not applicable

### Definition and Assessment of safety related events

### Reporting of Safety related events

# STATISTICAL METHODS

(ICH/E6 6.9; AGEK 8; SPIRIT # 14, 20)

Statistical considerations

ICH: A description of the statistical methods to be employed, including timing of any planned interim analysis(ses).

## Hypothesis

This is an explanatory study with no primary statistical hypothesis. The focus of the analysis will be on estimation.

## Determination of Sample Size

ICH: The number of subjects planned to be enrolled. In multicentre trials, the numbers of enrolled subjects projected for each trial site should be specified. Reason for choice of sample size, including reflections on (or calculations of) the power of the trial and clinical justification.

No formal sample size calculation was performed for this explanatory study that focuses on estimation rather than statistical hypothesis testing. The sample size is set to be approximately 1000 patients based on considerations regarding the recruitment potential and the size of published observational studies with similar objectives.

Events rates will be estimated in the entire population and in specific subgroups. Assuming that a subgroup includes 30% of the study sample (300 patients) the precision i.e. the half width of the 95% Clopper Person confidence interval for an event rate will be at most 5.8%.

The sample size is deemed adequate with respect to the main study objectives.

## Statistical criteria of termination of trial

ICH: A description of the "stopping rules" or "discontinuation criteria" for individual participants, parts of trial and entire trial.

No “stopping rules” have been defined for this observational study.

## Planned Analyses

ICH: A description of the statistical methods to be employed, including timing of any planned interim analysis(ses).

The analysis of this study will be exploratory and primarily make use of descriptive statistical methods. Confidence intervals and statistical tests will be used to highlight interesting aspects of the data.

Statistical significance will be declared for in our study is defined as p-values < 0.05. No correction for multiplicity will be applied.

Comparisions of event-rates from the present study with published data in the literature will also be performed .

### Datasets to be analysed, analysis populations

ICH: The selection of subjects to be included in the analyses (e.g., all randomized subjects, all dosed subjects, all eligible subjects, evaluable subjects).

The primary analysis population will include all patients fulfilling the inclusion/exclusion criteria (i.e. all eligible subjects). Subgroup analyses may be performed according for instance to the indication for craniotomy, site of intracranial lesion, time of extubation or need for unscheduled emergency computed tomography within 48 hours after index surgery.

### Primary Analysis

Analysis of early postoperative complication rate with need for unscheduled emergency computed tomography within 48 hours after index surgery and early operative revision (i.e. return to operating room). Event frequencies will be presented statistically and confidence intervals will be computed using the Clopper-Pearson method. The influence of the extubation time will be assessed in a logistic regression models including in addition other explanatory variables, such as the indication for craniotomy, site of intracranial lesion, age.

### Secondary Analyses

Analysis of mortality 30 days after index surgery.

Analysis of event rates (mortality, morbidity) in subgroups defined by the site of intracranial lesion, time of extubation or need for unscheduled emergency computed tomography within 48 hours after index surgery.

Analysis of the influence of need for unscheduled emergency computed tomography within 48 hours after index surgery and early operative revision on mortality.

### Interim analyses

ICH 6.9.1: ...... including timing of any planned interim analysis(ses).

An Interim analysis will be performed after 500 patients have completed the observational phase.

### Safety analysis

Not applicable, no interventions, no study drug, no study device involved for this trial.

### Deviation(s) from the original statistical plan

(ICH/E6 6.9.6)

ICH: Procedures for reporting any deviation(s) from the original statistical plan (any deviation(s) from the original statistical plan should be described and justified in protocol and/or in the final report, as appropriate).

Relevant deviations from the planned analysis will be mentioned and justified in the study report.

## Handling of missing data and drop-outs

(ICH/E6 6.9.5; AGEK 8.5; SPIRIT 20c)

ICH: Procedure for accounting for missing, unused, and spurious data.

No imputation of missing data will be performed for the primary analysis. Given the short follow-up period and the robust primary endpoint we do not expect a drop-out in the primary endpoint.

The primary analysis does not account for patients who were censored before 1 month. Therefore, survival models that account for censoring will be used for sensitivity analyses.

# QUALITY ASSURANCE AND CONTROL

(ICH/E6 6.11, 6.13; AGEK 12; SPIRIT #19, 23, 27)

ICH: Quality Control and Quality Assurance Procedures

## Data handling and record keeping / archiving

(KlinV, Art. 18, 45, 57, 62; ICH/E6 6.13; AGEK 12; SPIRIT #19, 27)

ICH: Data Handling and Record Keeping

All study relevant data will be assessed during the patients medical treatment by treating medical physicans and nurses. All data will be archived in the patients medical records. A Study nurse will transfer Data into the CRF.

### Case Report Forms

(ICH/E6 6.4.9)

ICH: The identification of any data to be recorded directly on the CRFs (i.e., no prior written or electronic record of data), and to be considered to be source data.

Study Data is recorded with paper Case Report Forms by a Study Nurse. For each enrolled study participant a CRF is maintained. Appropriate coded identification, e.g. participant number, sex and age are assessed for identification. A Study nurse will transfer Data into an electronic database (RedCap). Data will be transferred in RedCap in coded form (Coding will be continuing number). After coding, it will not be possible to identify patients as an individual in database (no identifying information e.g. name, date of birth, medical record number). Only study Team Members will have access to the coding list. Nicole Söll will store the Coding list till the end of the study. After termination of the study Coding list will be stored by Bettina Rotzetter (IT Managerin DKNS/ IT-Compliance Managerin BPRC).

### Specification of source documents

(ICH/E6 6.4.9)

ICH: The identification of any data to be recorded directly on the CRFs (i.e., no prior written or electronic record of data), and to be considered to be source data.

Source data is available at the site, which includes the original documents relating to the study, as well as the medical treatment and medical history of the participant. The patients file is source data and will be stored at the archive of the neurosurgery department.

### Record keeping / archiving

(ICH/E6 6.13)

ICH: Data Handling and Record Keeping

All study data will be archived for a minimum of 10 years after study termination or premature termination of the clinical trial.

## Data management

(ICH/E2; AGEK 12.2; SPIRIT #19)

### Data Management System

REDcap DMS, hosted by server infrastructure of the Dept. Head Organs and Neurology.

### Data security, access and back-up

Access is granted to study team using dedicated user accounts. Daily backups are stored to two different backup systems located on the Inselspital campus.

### Analysis and archiving

If requested, data can be archived in electronic vaults provided by the Dept. Head Organs and Neurology.

### Electronic and central data validation

The base system and functionality is first tested in identical testing/developing environment.

## Monitoring

(AGEK 12.1; SPIRIT #23)

There is no monitoring planned for this study.

## Audits and Inspections

(KlinV, Art. 58, 59; AGEK 12.1; SPIRIT #23)

Study documentation and the source data/documents are accessible to auditors/inspectors (also CEC and CA) and questions are answered during inspections. All involved parties keep the participant data strictly confidential.

## Confidentiality, Data Protection

(KlinV, Art. 18, 58; SPIRIT #27, 29)

Only involved parties in the study trial, Ethic Committee(s) and Swiss authorities will have direct access to source documents, protocols and datasets during and after the study.

## Storage of biological material and related health data

(KlinV, Art. 18; HVF Art. 28-32; SPIRIT #33)

Not applicable

# PUBLICATION AND DISSEMINATION POLICY

(ICH/E6 6.15)

ICH: Publication policy, if not addressed in a separate agreement.

The trial results will be communicated in form of abstracts and oral presentations at scientific meetings and publication of scientific papers. The core data within the database is used only for analysis and preparation of abstracts, presentation and writing of papers and articles. The database will not be shared outside of the department of neurosurgery at the Inselspital in Bern.

# FUNDING AND SUPPORT

(KlinV, Art. 25i; ICH/E6 6.14; SPIRIT #4)

## Funding

(KlinV, Art. 25i)

ICH: Financing and insurance if not addressed in a separate agreement.

The study will be funded through the the department of neurosurgery, Inselspital Bern.

## Other Support

(KlinV, Art. 25i)

ICH: Financing and insurance if not addressed in a separate agreement.

Not applicable

# INSURANCE

(KlinV Art 12, 13; ICH/E6 6.14, AGEK 10.3; SPIRIT #30)

ICH: ….and insurance if not addressed in a separate agreement.

Not applicable

# REFERENCES

(ICH/E6 6.2.7)

ICH: References to literature and data that are relevant to the trial, and that provide background for the trial.

Lin JP, Pay N, Naidich TP et al. Computed tomography in the postoperative care of neurosurgical patients. *Neuroradiology*. 1977;12(4):185-9.

1. Khaldi A, Prabhu VC, Anderson DE, Origitano TC: The clinical significance and optimal timing of postoperative computed tomography following cranial surgery. *J Neurosurg*. Published online December 18, 2009; DOI: 10.3171/2009.11. JNS081048.

Kondziolka D. The value of a postoperative computed tomography scan. *J Neurosurg*. published online December 18, 2009; DOI: 10.3171/2009.10.JNS091531.

1. Bruder N, Stordeur JM, Ravussin P et al. Metabolic and Hemodynamic Changes During Recovery and Tracheal Extubation in Neurosurgical Patients: Immediate Versus Delayed Recovery. Anesth Analg 1999;89:674-8.
2. Bruder NJ. Awakening management after neurosurgery for intracranial tumours. *Current Opinion in Anaesthesiology* 2002, 15:477-482.
3. Seifman MA, Lewis PM, Rosenfeld JV, Hwang PYK. Postoperative intracranial haemorrhage: a review. *Neurosurg Rev*. 2011 Oct;34(4):393-407. doi: 10.1007/s10143-010-0304-3. Epub 2011 Jan 19.
4. Lassen B, Helseth E, Ronning P, et al. Surgical Mortality at 30 Days and Complications Leading to Recraniotomy in 2630 Consecutive Craniotomies for Intracranial Tumors. *Neurosurgery* 68:1259-1269, 2011.
5. Taylor WAS, Thomas NWM, Welling JA, Bell BA. Timing of postoperative intracranial hematoma development and implications for the best use of neurosurgical intensive care. *J Neurosurg* 82:48-50, 1995.
6. Kalfas IH, Little JR. Postoperative hemorrhage: A survey of 4992 intracranial procedures. *Neurosurgery*.1988;23:343- 347.
7. Magni G, La Rosa I, Gimignani S et al. Early Postoperative Complications After Intracranial Surgery. *J Neurosurg Anesthesiol* 2007;19:229–234.
8. Jiang ZY, Allen K, Kutz JW, Isaacson B. Clinical Impact of Early CT Scans after Lateral Skull-Base Surgery. *Otolaryngol Head Neck Surg* 2013 Nov;149(5):786-8. doi: 10.1177/0194599813502311. Epub 2013 Sep 5.
9. Djian MC, Blanchet B, Pesce F et al. Comparison of the Time to Extubation After Use of Remifentanil or Sufentanil in Combination with Propofol as Anesthesia in Adults Undergoing Nonemergency Intracranial Surgery: A Prospective, Randomized, Double-Blind Trial. Clinical Therapeutics/Vol 28, Number 4, 2006.
10. Basali A, Mascha EJ, Kalfas I, Schubert A. Relation between perioperative hypertension and intracranial hemorrhage after craniotomy. *Anesthesiology* 2000;93:48-54.

Bilotta F, Caramia R, Paoloni FP et al. Early postoperative cognitive recovery after remifentanil-propofol or sufentanil-propofol anaesthesia for supratentorial craniotomy: a randomized trial. *Eur J Anaesthesiol*. 2007 Feb;24(2):122-7.

1. Bilotta F, Lam AM, Doronzino A et al. Esmolol blunts postoperative hemodynamic changes after propofol-remifentanil total intravenous fast-track neuroanesthesia for intracranial surgery. *J of Clinical Anesthesia* (2008) 20, 426-430.

# APPENDICES

ICH: (NOTE: Since the protocol and the clinical trial/study report are closely related, further relevant information can be found in the ICH Guideline for Structure and Content of Clinical Study Reports.)

1. Other

Case Report Form (e.g. CRF)

1. [↑](#endnote-ref-1)
