# Supplementary material for: No Routine Postoperative Head CT following Elective Craniotomy – A Paradigm Shift?
Source: PLoS One. 2016 Apr 14;11(4):e0153499. doi: 10.1371/journal.pone.0153499 (PMC4831779; doi:10.1371/journal.pone.0153499)
Supplement: S2 File — (PDF) [file pone.0153499.s002.pdf]

TREND Statement Checklist

Paper Section/ Topic	Item No	Descriptor	Reported? ✓	Pg #
**Title and Abstract**				
Title and Abstract	1	• Information on how unit were allocated to interventions	✓	2 Abstr.
		• Structured abstract recommended	✓	2
		• Information on target population or study sample	✓	2
**Introduction**				
Background	2	• Scientific background and explanation of rationale	✓	3 Intro.
		• Theories used in designing behavioral interventions	✓	3
**Methods**				
Participants	3	• Eligibility criteria for participants, including criteria at different levels in recruitment/sampling plan (e.g., cities, clinics, subjects)	✓	4 Meth.
		• Method of recruitment (e.g., referral, self-selection), including the sampling method if a systematic sampling plan was implemented	✓	4
		• Recruitment setting	✓	4
		• Settings and locations where the data were collected	✓	4-5
Interventions	4	• Details of the interventions intended for each study condition and how and when they were actually administered, specifically including:	✓	Methods
		○ Content: what was given?	✓	5-6
		○ Delivery method: how was the content given?	✓	5-6
		○ Unit of delivery: how were the subjects grouped during delivery?	✓	5-6
		○ Deliverer: who delivered the intervention?	✓	5-6
		○ Setting: where was the intervention delivered?	✓	5-6
		○ Exposure quantity and duration: how many sessions or episodes or events were intended to be delivered? How long were they intended to last?	✓	5-6
		○ Time span: how long was it intended to take to deliver the intervention to each unit?	✓	5-6
		○ Activities to increase compliance or adherence (e.g., incentives)	not applicable	
Objectives	5	• Specific objectives and hypotheses	✓	3 intro.
Outcomes	6	• Clearly defined primary and secondary outcome measures	✓	4
		• Methods used to collect data and any methods used to enhance the quality of measurements	✓	4-5
		• Information on validated instruments such as psychometric and biometric properties	✓	5
Sample Size	7	• How sample size was determined and, when applicable, explanation of any interim analyses and stopping rules	✓	4
Assignment Method	8	• Unit of assignment (the unit being assigned to study condition, e.g., individual, group, community)	✓	4
		• Method used to assign units to study conditions, including details of any restriction (e.g., blocking, stratification, minimization)	not applicable	
		• Inclusion of aspects employed to help minimize potential bias induced due to non-randomization (e.g., matching)	not applicable	

TREND Statement Checklist

Paper Section/ Topic	Item No	Descriptor	Reported? ✓	Pg #
**Title and Abstract**				
Title and Abstract	1	• Information on how unit were allocated to interventions	✓	2 Abstr.
		• Structured abstract recommended	✓	2
		• Information on target population or study sample	✓	2
**Introduction**				
Background	2	• Scientific background and explanation of rationale	✓	3 Intro.
		• Theories used in designing behavioral interventions	✓	3
**Methods**				
Participants	3	• Eligibility criteria for participants, including criteria at different levels in recruitment/sampling plan (e.g., cities, clinics, subjects)	✓	4 Meth.
		• Method of recruitment (e.g., referral, self-selection), including the sampling method if a systematic sampling plan was implemented	✓	4
		• Recruitment setting	✓	4
		• Settings and locations where the data were collected	✓	4-5
Interventions	4	• Details of the interventions intended for each study condition and how and when they were actually administered, specifically including:	✓	Methods
		○ Content: what was given?	✓	5-6
		○ Delivery method: how was the content given?	✓	5-6
		○ Unit of delivery: how were the subjects grouped during delivery?	✓	5-6
		○ Deliverer: who delivered the intervention?	✓	5-6
		○ Setting: where was the intervention delivered?	✓	5-6
		○ Exposure quantity and duration: how many sessions or episodes or events were intended to be delivered? How long were they intended to last?	✓	5-6
		○ Time span: how long was it intended to take to deliver the intervention to each unit?	✓	5-6
		○ Activities to increase compliance or adherence (e.g., incentives)	not applicable	
Objectives	5	• Specific objectives and hypotheses	✓	3 intro.
Outcomes	6	• Clearly defined primary and secondary outcome measures	✓	4
		• Methods used to collect data and any methods used to enhance the quality of measurements	✓	4-5
		• Information on validated instruments such as psychometric and biometric properties	✓	5
Sample Size	7	• How sample size was determined and, when applicable, explanation of any interim analyses and stopping rules	✓	4
Assignment Method	8	• Unit of assignment (the unit being assigned to study condition, e.g., individual, group, community)	✓	4
		• Method used to assign units to study conditions, including details of any restriction (e.g., blocking, stratification, minimization)	not applicable	
		• Inclusion of aspects employed to help minimize potential bias induced due to non-randomization (e.g., matching)	not applicable	

TREND Statement Checklist

Paper Section/ Topic	Item No	Descriptor	Reported? ✓	Pg #
**Title and Abstract**				
Title and Abstract	1	• Information on how unit were allocated to interventions	✓	2 Abstr.
		• Structured abstract recommended	✓	2
		• Information on target population or study sample	✓	2
**Introduction**				
Background	2	• Scientific background and explanation of rationale	✓	3 Intro.
		• Theories used in designing behavioral interventions	✓	3
**Methods**				
Participants	3	• Eligibility criteria for participants, including criteria at different levels in recruitment/sampling plan (e.g., cities, clinics, subjects)	✓	4 Meth.
		• Method of recruitment (e.g., referral, self-selection), including the sampling method if a systematic sampling plan was implemented	✓	4
		• Recruitment setting	✓	4
		• Settings and locations where the data were collected	✓	4-5
Interventions	4	• Details of the interventions intended for each study condition and how and when they were actually administered, specifically including:	✓	Methods
		o Content: what was given?	✓	5-6
		o Delivery method: how was the content given?	✓	5-6
		o Unit of delivery: how were the subjects grouped during delivery?	✓	5-6
		o Deliverer: who delivered the intervention?	✓	5-6
		o Setting: where was the intervention delivered?	✓	5-6
		o Exposure quantity and duration: how many sessions or episodes or events were intended to be delivered? How long were they intended to last?	✓	5-6
		o Time span: how long was it intended to take to deliver the intervention to each unit?	✓	5-6
		o Activities to increase compliance or adherence (e.g., incentives)	not applicable	
Objectives	5	• Specific objectives and hypotheses	✓	3 intro.
Outcomes	6	• Clearly defined primary and secondary outcome measures	✓	4
		• Methods used to collect data and any methods used to enhance the quality of measurements	✓	4-5
		• Information on validated instruments such as psychometric and biometric properties	✓	5
Sample Size	7	• How sample size was determined and, when applicable, explanation of any interim analyses and stopping rules	✓	4
Assignment Method	8	• Unit of assignment (the unit being assigned to study condition, e.g., individual, group, community)	✓	4
		• Method used to assign units to study conditions, including details of any restriction (e.g., blocking, stratification, minimization)	not applicable	
		• Inclusion of aspects employed to help minimize potential bias induced due to non-randomization (e.g., matching)	not applicable	
